# The Pandemic Influenza Preparedness (PIP) Framework: strengthening laboratory and surveillance capacities in the Western Pacific Region, 2014–2017

**DOI:** 10.5365/wpsar.2018.9.5.008

**Published:** 2020-12-28

**Authors:** Hitesh Chugh, Gina Samaan, Tatiana Resnikoff, Isabel Bergeri, Jennifer Barragan, Erica Dueger

**Affiliations:** a WHO Regional Office for the Western Pacific, Manila, Philippines.; b Pandemic Influenza Preparedness Secretariat, World Health Organization, Geneva, Switzerland.; c Global Influenza Programme, World Health Organization, Geneva, Switzerland.; d Centers for Disease Control and Prevention, Atlanta, United States of America.; e National Centre for Epidemiology and Population Health, Australian National University, Canberra, Australia.; * Sanofi Pasteur, Lyon, France.

The World Health Organization’s (WHO) Member States unanimously adopted the Pandemic Influenza Preparedness (PIP) Framework in May 2011. (*1*) The Framework has two aims: (1) to improve the sharing of influenza viruses with pandemic potential; and (2) to increase the access of developing countries to vaccines and other life-saving products during a pandemic. Implementing the PIP Framework enables Member States to meet their obligations under the International Health Regulations, or IHR (2005), (*2*) and advance implementation of public health emergency preparedness. The PIP Framework contributes to national and regional preparedness, alert and response priorities across all focus areas of the Asia Pacific Strategy for Emerging Diseases for Public Health Emergencies (APSED) framework. (*3*)

One key benefit of the PIP Framework is the Partnership Contribution (PIP-PC). (*4*) Annually, US$28 million is provided to WHO (*5*) by influenza vaccine, pharmaceutical and diagnostic manufacturers that use the Global Influenza Surveillance and Response System (GISRS) – a network of laboratories conducting surveillance of seasonal, pandemic and zoonotic influenza viruses. (*4*) PIP-PC funds complement investments from other sources and are used in synergy with national, regional, and global funding to strengthen preparedness capacities globally and in priority countries in the Western Pacific Region. From 2014 to 2017, PIP-PC funds were used according to the first high-level implementation plan (HLIP I) developed by WHO, in consultation with stakeholders, which focused on five areas of work: laboratory and surveillance, burden of disease estimation, regulatory capacity-building, risk communication and planning for deployment of pandemic products. (*5*) These areas of work where capacity building has been targeted form the foundation for an effective response not only to an influenza pandemic but for novel respiratory viruses (e.g. SARS-CoV-2). While there have been achievements in all five areas of work, (*6*) this paper provides an overview of implementation achievements in laboratory and surveillance capacities in the Region.

Between 2014 and 2017, in addition to other agencies’ contributions for influenza capacity building, US$8.6 million of PIP-PC funds was invested in the Region (*6*) for improving laboratory and surveillance capacities through detection of respiratory diseases due to a novel virus, monitoring influenza trends including through sentinel surveillance systems and strengthening GISRS and global collaboration through information and virus sharing. Using the country selection criteria established in HLIP I, the WHO Regional Office for the Western Pacific identified five countries to receive PIP-PC funds: (*5*) Cambodia, Fiji, the Lao People's Democratic Republic, Mongolia and Viet Nam. While other regional and global investments have played a foundational role in establishing these national capacities, PIP-PC investment into national preparedness have supplemented these programs and further strengthened core capacities for pandemic response. By 2017, laboratory and surveillance capacities had improved in these five priority countries as well as in the Region more broadly (**Table 1**). Criteria used to measure improvement were indicators established in HLIP I (e.g. the number of countries consistently reporting to FluID (*7*)); some are based on country self-reporting and others are extracted from WHO databases.

**Table 1:** Changes in the laboratory and surveillance capacities in the five countries (*1*) prioritized for PIP-PC in WHO’s Western Pacific Region, 2014 (*2*)–2017

Capacity category	Funds spent (million US$) (*3*)	Capacity (data source)	No. of countries (*4*)	
			2014	2017
**Detection**	**$5.1**	**1. National event-based surveillance system in place including available protocols, definitions and procedures (country self-report)**	**1**	**5**
		**2. Rapid Response Team established and trained in the past year (country self-report)**	**3**	**5**
**Monitoring**	**$1.8**	**3. Influenza-like illness surveillance was conducted, samples collected weekly and regularly sent to a laboratory (country self-report)**	**2**	**5**
		**4. Severe acute respiratory infection surveillance conducted, samples collected weekly and regularly sent to a laboratory (country self-report)**	**2**	**4**
		**5. Capacity for influenza virus sequencing (country self-report)**	**2**	**3**
		**6. Influenza surveillance reports with integrated data published in the public domain (country self-report)**	**2**	**3**
		**7. Consistently** (*6*) **reported virological data to WHO FluNET during the influenza season (WHO database)**	**4**	**5**
		**8. Consistently** (*6*) **reported epidemiological data to WHO FluID during the influenza season (WHO database)**	**0**	**5**
**Global collaboration**	**$1.7**	**9. Shared influenza viruses with WHO at least once a year in the last two years (WHO database)**	**5**	**5**
		**10. Staff trained and certified to ship influenza clinical specimens/virus isolates out of the country (WHO database)**	**2**	**4**
		**11. Participated yearly and scored 100% in the WHO External Quality Assessment Project for the detection of influenza viruses by real-time polymerase chain reaction in 2014–2017 (WHO database)**	**5**	**4**

1. Cambodia, Fiji, the Lao People's Democratic Republic, Mongolia and Viet Nam.

2. Funding period not implementation period.

3. Figures extracted from WHO financial management system on 7 March 2018, net of programme support costs.

4. Number of countries receiving PIP-PC funds.

5. Consistently means that a country reports weekly at least 60% of the weeks during the influenza season.

All PIP-PC priority countries improved influenza detection capacities through strengthened event-based surveillance (EBS) systems, (*8*) particularly at the human–animal interface (HAI) (see **Table 1**). All five also established or maintained their rapid response teams. These teams are integral for countries to rapidly identify and control disease outbreaks, as noted by the IHR (2005). Examples of how countries have improved their capacities were recently reported in IHR Joint External Evaluation missions. (*9*-*12*) Activities that supported these achievements included provision of technical assistance for influenza surveillance protocols and guidelines and delivery of training programs in field epidemiology, HAI rapid response and surveillance. These programmes have been integral for countries in rapidly identifying and investigating potential outbreaks, highlighting the impact of the investments on broader country-level preparedness and strengthening of core capacities under the IHR (2005) using the Asia Pacific Strategy for Emerging Diseases and Public Health Emergencies (APSED III) as an implementing framework.

All five PIP-PC recipient countries conduct epidemiological and virological surveillance for influenza in either outpatient or inpatient populations (**Table 1**). All five countries have the improved capacity to routinely monitor influenza virological and epidemiological trends; three have published surveillance reports that integrate virological and epidemiological findings. Furthermore, all five countries consistently share influenza viruses/specimens with GISRS and report surveillance data to the WHO reporting platforms FluNet (*13*) and FluID. To enhance regional information sharing, the WHO Regional Office for the Western Pacific Region launched an online influenza dashboard that presents consolidated seasonal and avian influenza surveillance data. (*14*) It provides public access to regularly updated regional influenza activity and provides an opportunity for countries to share important severity assessment evaluations during unusual outbreaks. Combined with virological data, this enables timely global situational monitoring and risk assessment and provides a collateral benefit to partners using GISRS.

Improvements in national influenza laboratory systems assure quality contribution to GISRS, thereby contributing to global preparedness for future influenza pandemics. PIP-PC funds helped support trainings in the five priority countries, and other countries in the Region as needed, on specimen collection and handling, virus isolation, molecular diagnostic techniques, sequencing and bioinformatics and laboratory biosafety and biosecurity. Supplies and equipment were provided to national laboratories to facilitate these capabilities. Regional technical experts routinely provided or sourced expertise to support and mentor influenza laboratory and epidemiology surveillance staff. Countries continue to participate in the WHO External Quality Assessment Project (*15*) for the detection of influenza viruses by polymerase chain reaction, and the results indicate ongoing capacity-strengthening needs.

There was a marked improvement in preparedness in the PIP priority countries from 2014 to 2017, including enhanced capacities to detect and respond to influenza-related events through improved EBS and laboratory capacities. In addition, improved indicator-based surveillance systems (*8*) allow estimation of influenza disease burden in support of high-risk group vaccination policies, as well as establishment of thresholds to monitor seasonality and severity. Meanwhile, improved cross-sectoral information sharing between public and animal health authorities facilitates risk assessment for public health action. These gains are attributed to the collective effort and commitment of national authorities, in parallel with investments made by national and international partners, including the PIP-PC funds and APSED III.

Investing in preparedness is an ongoing requirement. (*16*) There is a need for continuous support in surveillance and laboratory strengthening, particularly for laboratory capacity. Of the 27 countries and areas in the Western Pacific Region, 14 countries report to FluNet, 18 countries report to FluID and 15 countries routinely share influenza viruses with GISRS, so there are still opportunities for improvement. With the end of HLIP I implementation, further improvements in pandemic preparedness can build on the success achieved and the lessons learnt. While HLIP I has a sustainability principle, WHO put in further measures in HLIP II so that procurement and activity implementation is done with sustainability in mind.

In 2018, WHO launched HLIP II, which sets out an ambitious agenda for continuing to strengthen pandemic influenza preparedness capacities in 2018–2023. (*17*) A sixth area of work focusing on pandemic influenza preparedness planning was introduced to link the other five areas together. It provides an opportunity for capacity building efforts from HLIP I to be linked to the broader development and testing of national pandemic plans and health security strategies through simulation exercises and after-action reviews. In the Western Pacific Region, HLIP II implementation will increase focus on strengthening regional and national capacities in influenza risk, severity assessment and pandemic planning capacities. Following a successful initiation, the PIP Framework remains an effective tool to strengthen regional and country pandemic preparedness, particularly in the surveillance and laboratory area.
